# Rhythmic expression of the *cycle *gene in a hematophagous insect vector

**DOI:** 10.1186/1471-2199-7-38

**Published:** 2006-10-27

**Authors:** Antonio CA Meireles-Filho, Paulo R Amoretty, Nataly A Souza, Charalambos P Kyriacou, Alexandre A Peixoto

**Affiliations:** 1 Department of Biochemistry and Molecular Biology, Instituto Oswaldo Cruz – Fiocruz, Rio de Janeiro 21045-900, Brazil; 2 Department of Entomology, Instituto Oswaldo Cruz – Fiocruz, Rio de Janeiro 21045-900, Brazil; 3 Department of Genetics, University of Leicester, Leicester, LE1 7RH, UK

## Abstract

**Background:**

A large number of organisms have internal circadian clocks that enable them to adapt to the cyclic changes of the external environment. In the model organism *Drosophila melanogaster*, feedback loops of transcription and translation are believed to be crucial for the maintenance of the central pacemaker. In this mechanism the *cycle *(or *bmal1*) gene, which is constitutively expressed, plays a critical role activating the expression of genes that will later inhibit their own activity, thereby closing the loop. Unlike *Drosophila*, the molecular clock of insect vectors is poorly understood, despite the importance of circadian behavior in the dynamic of disease transmission.

**Results:**

Here we describe the sequence, genomic organization and circadian expression of *cycle *in the crepuscular/nocturnal hematophagous sandfly *Lutzomyia longipalpis*, the main vector of visceral leishmaniasis in the Americas. Deduced amino acid sequence revealed that sandfly *cycle *has a C-terminal transactivation domain highly conserved among eukaryotes but absent in *D. melanogaster*. Moreover, an alternative form of the transcript was also identified. Interestingly, while *cycle *expression in *Drosophila *and other Diptera is constitutive, in sandflies it is rhythmic in males and female heads but constitutive in the female body. Blood-feeding, which causes down-regulation of *period *and *timeless *in this species, does not affect *cycle *expression.

**Conclusion:**

Sequence and expression analysis of *cycle *in *L. longipalpis *show interesting differences compared to *Drosophila *suggesting that hematophagous vector species might present interesting new models to study the molecular control of insect circadian clocks.

## Background

A diversity of organisms, ranging from bacteria to humans, shows circadian rhythms in physiology and behavior that are controlled by endogenous oscillators. In mammals and flies, the core clocks are generated by two negative feedback loops that are interconnected to the same two positive basic helix-loop-helix (bHLH)/PAS-containing transcription factors CLOCK (CLK) and CYCLE (CYC) (also called BMAL1)(reviewed in [1,2]).

In *D. melanogaster*, CLK and CYC form a heterodimer that binds to upstream E-box sequences (*CACGTG*) in *period *(*per*) and *timeless *(*tim*), which in turn control their own expression by negatively regulating CLK/CYC mediated activation [1,2]. In the second loop, the products of *vrille *(*vri*) and *PAR domain protein 1 epsilon *(*Pdp1*ε), which are also activated by CLK/CYC, regulate *Clk *transcription by competing for the same site in its promoter. Whereas VRI represses *Clk *production just after lights off, PDP1ε activates it in the middle of the night, separating the phases of *Clk *transcription and repression [3,4]. These oscillations of gene expression and posttranslational regulation are necessary for the robustness and accuracy of overt physiological and behavioral rhythms.

Although the core clock molecules are relatively conserved between mammals and *D. melanogaster*, there are some interesting differences in, for example, the transcriptional control of *Clk *and *cyc *expression. In the suprachiasmatic nuclei (SCN) of mammals (where the central pacemaker is located), *Clk *is constitutively expressed [5] and *Bmal1 *is rhythmic, reaching its maximum abundance at dawn [6,7]. In contrast, *cyc *is constitutively expressed in *D. melanogaster *heads [8,9], while *Clk *shows rhythmic expression on the mRNA level, peaking during the night-day transition (ZT 22-2) [10,11]. Although the *Drosophila *CLK protein has also been reported to cycle with the same phase of its mRNA [10,11], recent data indicates that was a result of a methodological artifact [12,13]. Its ability to bind E-boxes and activate transcription in a cyclic manner in fact resides in its phosphorylation pattern, with only the late day/early night hypophosphorylated forms being capable of promoting *per *and *tim *expression [12,13].

The molecular study of circadian rhythms in insect vectors is still in its infancy. In sandflies the circadian expression profiles of *per*, *tim *and *Clk *has been studied in *Lutzomyia longipalpis*, the main vector of visceral leishmaniasis in the Americas [14]. While *per *and *tim *cycle as in other insects, peaking around ZT 13 [15-17], *Clk *expression peaks around ZT 9–13, about half a day later than in *D. melanogaster *[10,11,14]. This difference in *Clk *expression is correlated with differences in locomotor activity. *Drosophila *shows a bimodal/diurnal pattern, whereas *Lutzomyia *is predominately unimodal/nocturnal [14]. In addition, blood feeding causes a reduction in sandfly locomotor activity that is accompanied by a reduction in *per *and *tim*, but not *Clk *levels [14]. Thus, as the *Clk *profile of *L. longipalpis *is different from that of *D. melanogaster*, we wondered if the same would occur for its partner *cyc*.

We therefore cloned the *L. longipalpis cyc *gene and report here its genomic structure and the putative amino acid sequence. The presence of an alternative transcript is also identified. In addition we have analyzed the daily expression of *cyc *in males and females, as well as its expression after a blood meal.

## Results

### Cloning and sequence analysis

**Figure 1:**
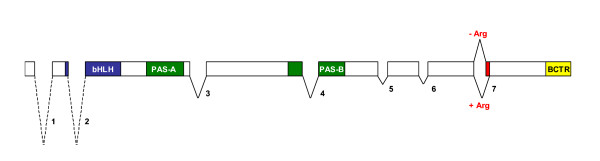
**Genomic structure *L. longipalpis cycle *gene**. Schematic representation of the genomic structure *L. longipalpis cycle *gene. The introns are numbered and were drawn to scale. The first and the second introns are depicted by a broken line to indicate that their complete sequence has not been determined. The coding region of the *L. longipalpis cycle *gene consists of 8 exons and 7 introns and the conserved motifs are shown in blue (bHLH), green (PAS A and B respectively) and yellow (BCTR). In red is marked the approximate position of the alternatively spliced Arg (see text).

The sequence of the *Lutzomyia longipalpis cycle *gene (GenBank accession number DQ841151) was obtained using a PCR gene walking approach. An initial fragment was obtained using degenerate primers. Based on this first sequence new specific primers were designed and used in new reactions to obtain further sequences. The 5' and 3' ends were obtained using RACE techniques (see Methods for details). Introns were mapped by comparing PCR fragments generated using cDNA and genomic DNA as templates. Fig 1 shows a schematic representation of the *L. longipalpis cyc *gene with its seven introns. The figure also shows the regions coding for the different domains of the protein and the position of an alternative splice form (see below).

**Figure 2:**
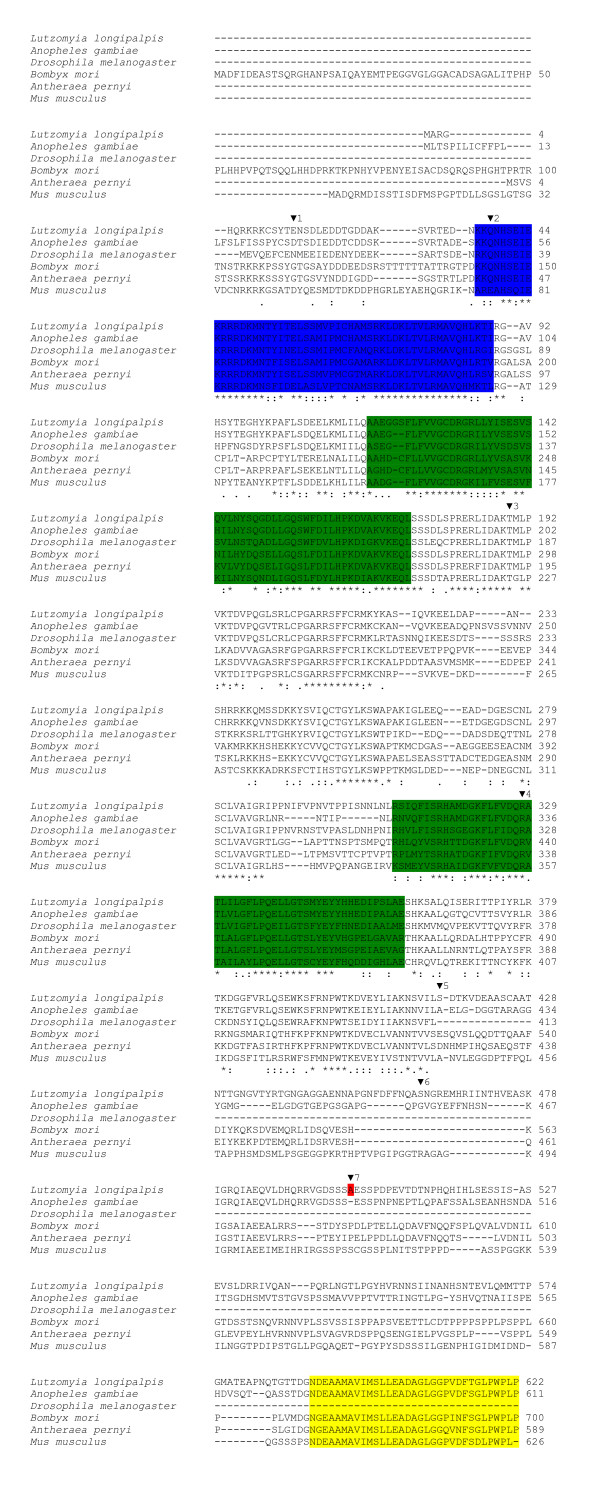
**Alignment of CYCLE protein sequences**. Protein alignment of the *L. longipalpis *CYCLE sequence with orthologues from *Anopheles gambiae *(Ensembl: ENSANGP00000027387), *Drosophila melanogaster *(GenBank™: AAC39124), *Bombyx mori *(GenBank™: BAB91178), *Antheraea pernyi *(GenBank™: AAR14937) and *Mus musculus *(GenBank™: AAH25973). The marked motifs use the same colors as in Fig 1.

**Figure 3:**
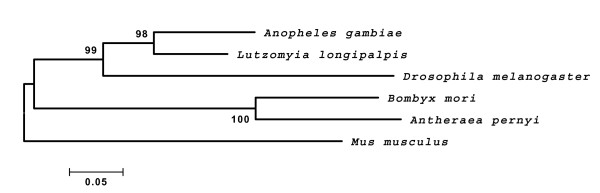
**Neighbor-joining tree of CYCLE protein sequences**. Neighbor-joining tree using the alignment of the protein sequences shown in Fig 2, complete deletion and Poisson corrected distances. *Mus musculus *was used as the outgroup.

*L. longipalpis cycle *codes for a putative protein of 622 amino acids homologous to CYC from other species (Fig 2). Alignment of the sandfly CYC sequence with insect orthologues (*Anopheles gambiae*, *D. melanogaster*, *Bombyx mori *and *Antheraea pernyi*) and one mammal (*Mus musculus*) reveals high conservation in some particular regions such as the bHLH DNA binding domain and the protein dimerization PAS A and PAS B regions, especially when compared to *A. gambiae *and *D. melanogaster *(see Table 1). In addition, we were able to find in sandfly CYC the BMAL1 C-terminal region ("BCTR"), which was characterized as an activation of the CLK/BMAL1 heterodimer in a mammalian cell culture [18] (Fig 2). This region in *L. longipalpis *CYC is 96.87% identical to the mosquito predicted CYC sequence, 87.5% identical to the moth *A. pernyi *and 93.75% identical to the mouse BMAL1 sequences (Table 1). Interestingly, this motif is not present at all in *Drosophila *(Fig 2; see Discussion). Fig 3 shows a Neighbor-joining tree using the alignment of the protein sequences shown in Fig 2. As expected *L. longipalpis *CYC clustered with the *A. gambiae *sequence.

The approximate positions of the seven introns of the *L. longipalpis cycle *gene are also marked in fig 2 by inverted triangles. Inspection of cDNA and genomic sequences available for *Anopheles gambiae cyc *revealed that only three out of the seven intron positions of *L. longipalpis cyc *(2, 3 and 7) are conserved between the two species (data not shown). Comparison of different cDNA sequences also revealed the existence of a rare alternative splice transcript missing only one Arginine codon (Fig 1 and 2). Nevertheless this single difference potentially alters the ability of the putative protein to be phosphorylated (see below). This minor transcript corresponds to about 20% of all sequenced cDNA fragments (7/35).

**Figure 4:**
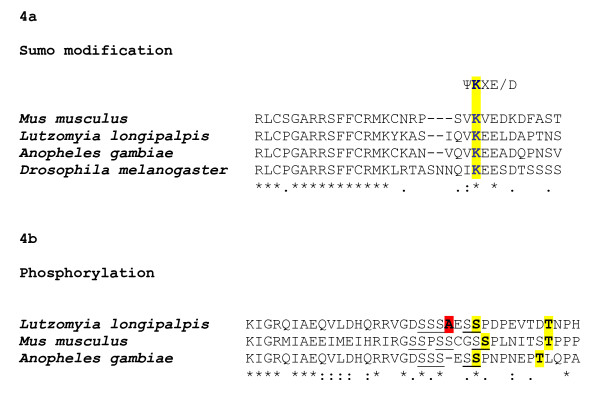
**Potential Sumo modification and Phosphorylation sites in the *L. longipalpis *CYCLE sequence**. Potential Sumo modification and Phosphorylation sites in the *L. longipalpis *CYCLE sequence. (A) Alignment of the *M. musculus*, *A. gambiae*, *D. melanogaster *and *L. longipalpis *PAS linker region. The three insect species listed contains a potential SUMO consensus motif and the correspondent Lys that is sumoylated in mammals. (B) A Ser-rich region (Ser underlined) is shown for *M. musculus*, *A. gambiae *and *L. longipalpis *CYC. Ser and Thr residues are phosphorylated in mammals and their potential cognates in *L. longipalpis *and *A. gambiae *CYC are highlighted in yellow. In red is marked the position of the alternatively spliced Arg (see text).

We also searched in sandfly CYC sequence possible conserved residues for pos-translational modification (Fig 4a,b). We were able to find a lysine at the position Lys-225 in a conserved sumo consensus site (ΨKXE/D, where Ψ is a hydrophobic residue and X may be any amino acid) in the PAS link region, at an approximate position where its mammalian homologue is sumoylated *in vivo *(Lys-259) [19] (Fig 4a). In addition we were able to find Ser-502 and a Thr-510 at conserved positions, corresponding to the mammalian Ser-527 and Thr-534, which are phosphorylated *in vitro *[20]. This region was further examined for potential kinase substrate sites using Scansite 2.0 (stringency levels = high) [21] and the Ser-502 was identified as potential casein kinase-1 phosphorylation site (Score: 0.3474; Percentile: 0.081%) (Fig 4b). Interestingly, the rare alternative transcript identified as missing a single Arg did not reach significance in the phosphorylation prediction at high stringency, suggesting another level of sandfly CYC regulation.

### Temporal *cyc *expression analysis

As *per *mRNA levels are differentially expressed between the head and body in females of *D. melanogaster*, female sandfly heads and bodies were analyzed separately. Sandfly males were not dissected since no differences are observed in *per *expression between heads and bodies in *Drosophila *[22].

**Figure 5:**
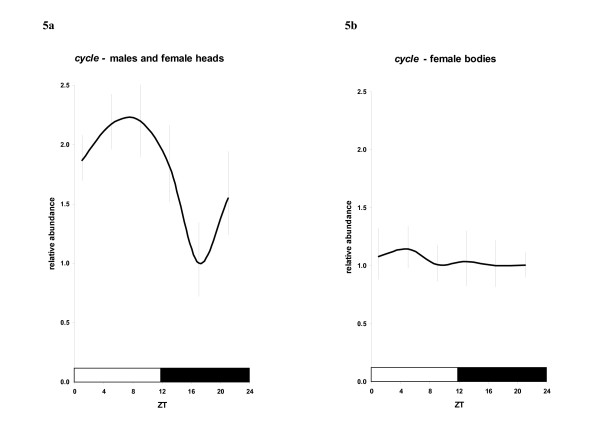
**Daily expression of *L. longipalpis cycle***. Daily expression of *L. longipalpis cycle*. A. Pooled data for males and female heads. B. Female bodies. Bars represent the mean relative abundance +/- the range based on the SEM (Standard Error of the Mean).

Analysis of *L. longipalpis cyc *expression in males and female heads under LD12:12 relative to the *rp49 *constitutive control indicates clear cycling in mRNA abundance. As for sandfly *per*, *tim *and *Clk *[14] no significant differences between males and female heads were observed, and therefore these results were pooled. ANOVA indicates that *cyc *mRNA levels are significantly different among ZT groups (F_5,72 _= 4.481; P < 0.001) with a peak around ZT 5–9, with levels over two-fold higher compared to the levels at ZT 17 (P < 0.001, LSD – Least significant difference analysis) (Fig 5a). This is similar to the observed *Bmal1 *cycling in mammals [6,7] but different compared to most insects analyzed to date, where its expression is constitutive [8,9,16,23]. On the other hand, in female bodies, *cyc *expression was constitutive (F_5,34 _= 0.086; P = 0.994) (Fig 5b), in contrast to *Bmal1 *expression in all mammalian tissues analyzed so far [24,25].

### *cyc *expression analysis in blood-fed females

**Figure 6:**
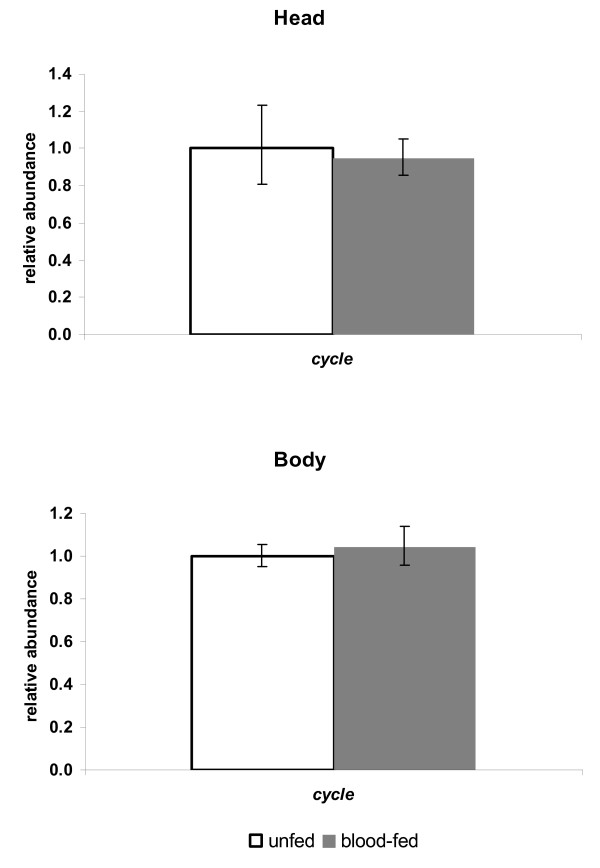
***cycle *expression in unfed and blood-fed *L. longipalpis *females**. Relative expression of *cycle *in unfed and blood-fed *L. longipalpis *female heads (top) and bodies (bottom). Columns (+/- the range based on the SEM) represent the normalized relative abundance.

In order to know if blood feeding has an effect on *cyc *expression as it does for *per *and *tim *[14], we assayed its mRNA levels 27 hs after a full blood meal. But, as for *Clk *[14], *cyc *levels were not significantly altered in heads and bodies of ingurgitated females compared to unfed controls (Fig 6).

## Discussion

In this study we characterized the sequence, genomic structure and expression of the *cycle *gene in the hematophagous sandfly *L. longipalpis*. Analysis of predicted protein sequence revealed its homology with *cyc *from others species (Fig 2). Interestingly, the BMAL1 C-terminal region ("BCTR"), which was characterized as responsible for the activation of the CLK/BMAL1 heterodimer in a mammalian cell culture [18], was also found in sandfly CYC. The conservation of this region in all animals analyzed so far (except *Drosophila*) suggests that sandfly CYC may also possess a C-terminal transactivation domain [8,23,26,27]. Chang et al [26] studying moth clock genes have suggested that the BCTR is very ancient, being lost in *Drosophila *CYC probably because it became redundant after the fruitfly CLK had acquired a new transactivation domain, a large poly-Q region. This latter domain is not found in the moth CLK orthologue and we are currently cloning the sandfly *Clk *to determine if the same is true for this vector species.

An important feature of mammalian CYC regulation is the phosphorylation and sumoylation of its serine/threonine and lysine residues respectively [19,20,28]. Aligning CYC homologues from different species we were able to find a lysine in the PAS link region of sandfly CYC at an approximately similar position where its homologue in mammals is sumoylated (Fig 4a). In addition, prediction phosphorylation site analysis identified Ser-502 as a potential target for posttranslational modification, but only in the more abundant form. In the alternative transcript identified, the missing Arg alters the ability of the Ser to be phosphorylated. This difference is noteworthy since in mammals only the hypophosphorylated form is able to bind to E-boxes *in vitro*, showing that phosphorylation of BMAL1 might play an important role in pacemaker regulation [20,28]. Taken together, these results suggest that sandfly CYC might be regulated at different levels (transcriptional and posttranslational), which may be important for its role in the sandfly pacemaker.

Our results on daily gene expression in males and female heads, unexpectedly, resemble data from mammals where *cyc *expression is also rhythmic (Fig 5a) [6,7,24]. Unlike most insects analyzed so far (where no oscillation of *cyc *mRNA was detected [9,16,23], but see Rubin et al [27]) sandfly *cyc *cycled robustly, beginning to rise at the end of the night (ZT 21) and peaking in the middle of the day ZT 5–9 (Fig 5a).

In *Drosophila *posttranslational mechanisms are necessary to provide optimal levels and subcellular localization of clock proteins. Earlier data have indicated that *per *and *tim *start to accumulate when CLK levels are decreasing [10,11], and this cannot be satisfactorily explained by a simple feedback loop model [1,2]. This contradiction was recently clarified by two papers that show that CLK levels in fact do not cycle [12,13]. Nevertheless, CLK transcriptional activity is rhythmic, via its phosphorylation levels. While hyperphosphorylated CLK predominates during times of transcriptional repression (late night/early morning), hypophosphorylated CLK is more abundant during times of transcriptional activation (late day/early night) [12,13]. The authors of these studies suggest that hypophosphorylated CLK forms complexes with CYC at midday, bind to E-boxes and initiate *per *and *tim *transcription. Once the TIM/PER/DBT complex enters the nucleus it represses transcription by inhibiting CLK/CYC E-box binding and promoting CLK hyperphosphorylation and degradation [12,13]. On the other hand our previous report on *per*, *tim *and *Clk *expression in sandflies satisfied a simple feedback loop model, since *per *and *tim *levels rise at the time when *Clk *levels reaches its peak [14]. Given that in head oscillators *cyc *expression is earlier than *Clk *and that we identified at least one strong putative motif for phosphorylation, we propose that sandfly CYC might be subject to posttranslational modification, which would provide the necessary time delay for its accumulation at the appropriate time of day (ZT 13, when it can dimerize with the product of *Clk *and drive *per *and *tim *transcription [9,14]).

In contrast to heads, *cyc *expression in female bodies was shown to be constitutive (Fig 5b). In *Drosophila per *was shown to be constitutively expressed in ovaries [22] causing a strong damping in *per *cycling in female bodies. In fact, sandfly *per *is also constitutive in female bodies [14]. The differential regulation of *cyc *through the sandfly body suggests that, as in *Drosophila *and mammals [29], clock genes in *L. longipalpis *may also play different roles in different tissues, reflecting particular interactions with different molecules, what would finally lead to the coordination of other aspects of sandfly physiology. Interestingly the mammalian orthologue BMAL1 was shown to interact with non-circadian transcription factors, which in turn could respond to different kinds of stimuli [30].

Finally, data on blood-fed females shows that, although *per *and *tim *expression are downregulated, *Clk *and *cyc *are not [[14] and this report]. Since the latter two are activators of the formers, we believe that blood-feeding might regulate negatively CLK and CYC function at the posttranscriptional level, leading to diminished *per *and *tim *activation. This could be mediated by changes in NAD(P)H/NAD(P)+ levels, which can be altered by blood-feeding in other insect species [31,32]. Furthermore, changes in redox state have been observed to alter mammalian CLK activity *in vitro *[33]. This latter observation is consistent with the observations that feeding and fasting, which would be expected to change the redox profile, can entrain mammalian peripheral clocks independently of the LD cycles [34,35]. However, restricted-feeding regimes in *Drosophila *do not appear to influence circadian behavior or molecular rhythms of *per *and *tim *[36].

## Conclusion

The present results, together with our previous data, show that the molecular clock of *L. longipalpis *shows interesting differences compared to *Drosophila*, suggesting that blood-sucking insect vector species might present very interesting comparative models to study circadian rhythms and its molecular control. In addition, since the circadian clock drives activity and feeding behavior in insect vectors, understanding the molecular machinery of the clock may add important information in the dynamics of vector-borne disease transmission.

## Methods

### Insects

*L. longipalpis *sandflies from a Lapinha (Minas Gerais State, Brazil) laboratory colony were reared as previously described [14,37]. Briefly, for the temporal gene expression experiments three independent replicate samples with circa 40 sandflies were collected on the fourth day of entrainment at ZTs 1, 5, 9, 13, 17 and 21. Only females were dissected due to their differential pattern of expression between heads and body tissues [14]. For blood-feeding experiments two to three-day-old females were blood-fed on an anaesthetized hamster during 10 min at the light-dark transition. Afterwards blood-fed and unfed controls (from the same cage) were separated and kept in different cages in an incubator at 25°C and LD12:12. Since blood-fed and unfed controls had to be visually separated after the feeding period, they were subjected to a phase-delay of 2 h, that is, placed in a different incubator with lights turning on and off 2 h later than the previous one where they were entrained. They were collected and frozen at ZT 13 in the following day (27 h after the blood meal – 2 h needed to separate blood-fed and unfed controls plus 25 h to reach the ZT 13 in the next day). This procedure was shown not to affect sandfly behavior nor *per*, *tim *and *Clk *expression [14].

### Cloning of sandfly *cyc*

Genomic sandfly DNA from circa 20 individuals was extracted with the GenomicPrep™ (Amersham Biosciences) kit according to manufacturer instructions. A fragment homologous to the *Drosophila cyc *was first amplified from *L. longipalpis *genomic DNA using the degenerate primer PCR technique. The primer sequences were as follows: 5'CYCdeg1, 5' *A(A, G)(A, C)GN(A, C)GN(A, C)GNGA(T, C)AA(A, G)ATGAA *3' & 3'CYCdeg1, 5' *AC(C, T)TTNCC(A, G, T)AT(A, G)TC(C, T)TTNGG(A, G)TG *3'. Sequential reactions were carried out to reach the 3' and 5' end of the gene as follows. For the missing 5' of the gene we used the "5' Race System for Rapid Amplification of cDNA Ends" kit (Gibco BRL). Primer used in the 1^st ^strand synthesis 3'llCYCexp1: 5' *TTATGGAAGTGGCCATGGGAGTCC *3'. Then the first PCR reaction was done with the primers 5'RACE AAP: 5' *GGCCACGCGTCGACTAGTACGGGIIGGGIIGGGIIG *3' & 3'llCYC8: 5' *CTCCTTGACCTTAGCCACATC *3'. Reamplification of this material was done with the nested AUAP 5'*GGCCACGCGTCGACTAGTAC *3' & 3' llCYC7 5' *TGGGAGTAATTGAGGACCTGC *3' primers according to manufacturer instructions. For the 3' region a preliminary reaction with specific and degenerate primers was done before the 3'RACE: initial reaction with primers 5'llCYC2 5' *GGTCCTCAATTACTCCCAAG *3' & 3'CYCdeg2 5' *TTCATNC(G, T)(A, G)CA(A, G)AA(A, G)AA *3' and later with the primers 5'llCYC3 5' *CAATGCTTCCGGTGAAGACG *3' & 3' CYCdeg3 5' *(G, C)(A, T)NGTNCCNA(A, G)(A, G, T)AT(C, T)TC(C, T)TG *3'. The 3' extreme end of the gene was obtained with the following primers: 5'llCYC7 5' *CAGTTCATCTCTCGTCATGCC *3' & oligo dT and later a nested reaction: 5'llCYC6 5' *CGTTGATTCTGGGCTTCCTAC *3' & oligo dT. Gene fragments were cloned in a pMOS vector (Amersham Biosciences) and sequenced at the Department of Biochemistry and Molecular Biology, Instituto Oswaldo Cruz – FIOCRUZ on an ABI 377XL DNA analyzer using BigDye Terminator v3.0 (Applied Biosystems). Sequence analysis was performed with the GCG software and the NCBI website [38]. Potential phosphorylation sites were detected using Scansite 2.0, with high stringency levels [21]. The sandfly *cyc *sequence was submitted to the GenBank under the accession number DQ841151.

### Quantitative RT-PCR

Firstly, mRNA was extracted with the QuickPrep™ Micro mRNA Purification kit (Amersham Biosciences) and reverse-transcribed with the TaqMan Reverse Transcription Reagents (Applied Biosystems) using the oligo-dT primer according to manufacturer instructions. Levels of *cyc *mRNA relative to non-cycling levels of *rp49 *were assayed by quantitative Real Time PCR using an ABI PRISM^® ^7000 (Applied Biosystems) as previously described [14]. We used 3 different sets of primers for *cyc *and one for *rp49*. *cyc *primer pairs: 5' *TGCCAAAACAATGCTTCCGG *3' & 5' *ACGTTGCCCTTTGATCGACA *3'; 5' *AATTGATGCCAAAACAATGC *3' & 5' *AGAATCAACGTTGCCCTTTG *3'; 5' *GATGCCAAAACAATGCTTCC *3' & 5' *GTGCCCAGGACTTGAGGTAG *3'. *rp49 *primer pair: 5' *CGATATGCCAAGCTAAAGCA *3' & 5' *GGGCGATCTCAGCACAGTAT *3'. At least one of each primer in the pair spanned an exon/intron boundary to prevent amplification from any genomic DNA contamination. Indeed, melting-temperature curves showed a single amplified product and the absence of primer-dimer formation, which was confirmed by gel electrophoresis (data not shown). Non-template controls were included for each primer pair to check for any significant levels of contaminants. Standard curves were used to confirm that primers pairs had similar reaction efficiencies. Reactions were carried out in quadruplicates in a final reaction volume of 30 μl using 2× SYBR^® ^Green PCR Master Mix (Applied Biosystems) and primers at a final concentration of 500 nM. Amplifications were carried out for 50 cycles as follows: (i) 95°C, 10 sec; (ii) 60°C, 60 sec; (iii) 78°C, 30 sec (florescence recorded); (iv) repeat. Raw data were exported to EXCEL (Microsoft) for analysis.

## Abbreviations

Bmal1, Brain and muscle Arnt-like protein-1; SCN, suprachiasmatic nuclei; qRT-PCR, quantitative Reverse Transcription – Polymerase Chain Reaction; ZT, zeitgeber time; LD, light-dark; bHLH-PAS, basic helix-loop-helix-Per-Arnt-Sim.

## Authors' contributions

ACAMF carried out most of experiments and drafted the manuscript. PRA did part of the cloning steps and sequencing. NAS helped in the acquisition of sandfly samples and to design the blood-feeding experiment. CPK participated in the coordination, helped to write the manuscript and supervised ACAMF during his stay in Leicester. AAP is the principal investigator, participated in its design and coordination, and helped to write the manuscript. All authors read and approved the final manuscript.

## Figures and Tables

**Table 1:** Percentage identities between the *Lutzomyia longipalpis *CYCLE and its orthologues in some other organisms in the whole protein (excluding regions with gaps) and in some particular domains

	Total	bHLH	PAS A	PAS B	BCRT
*Anopheles gambiae*	84.97	94.34	92.73	92.31	96.87
*Drosophila melanogaster*	67.36	84.91	80.00	69.23	-
*Bombyx mori*	60.36	83.02	76.36	65.38	87.50
*Antheraea pernyi*	60.62	84.91	72.73	63.46	87.50
*Mus musculus*	62.18	73.58	76.36	69.23	93.75
